# Exchange-biasing topological charges by antiferromagnetism

**DOI:** 10.1038/s41467-018-05166-9

**Published:** 2018-07-17

**Authors:** Qing Lin He, Gen Yin, Alexander J. Grutter, Lei Pan, Xiaoyu Che, Guoqiang Yu, Dustin A. Gilbert, Steven M. Disseler, Yizhou Liu, Padraic Shafer, Bin Zhang, Yingying Wu, Brian J. Kirby, Elke Arenholz, Roger K. Lake, Xiaodong Han, Kang L. Wang

**Affiliations:** 1 Department of Electrical and Computer Engineering, Department of Physics and Astronomy, Department of Materials Science and Engineering, University of California, Los Angeles, CA 90095 USA; 2 International Center for Quantum Materials, School of Physics, Peking University, Beijing, 100871 China; 3 NIST Center for Neutron Research, National Institute of Standards and Technology, Gaithersburg, MD 20899-6102 USA; 4 Department of Electrical and Computer Engineering, University of California, Riverside, CA 92521-0204 USA; 5 Advanced Light Source, Lawrence Berkeley National Laboratory, Berkeley, CA 94720 USA; 6 Beijing Key Lab of Microstructure and Property of Advanced Materials, Beijing University of Technology, Beijing, 100124 China

## Abstract

Geometric Hall effect is induced by the emergent gauge field experienced by the carriers adiabatically passing through certain real-space topological spin textures, which is a probe to non-trivial spin textures, such as magnetic skyrmions. We report experimental indications of spin-texture topological charges induced in heterostructures of a topological insulator (Bi,Sb)_2_Te_3_ coupled to an antiferromagnet MnTe. Through a seeding effect, the pinned spins at the interface leads to a tunable modification of the averaged real-space topological charge. This effect experimentally manifests as a modification of the field-dependent geometric Hall effect when the system is field-cooled along different directions. This heterostructure represents a platform for manipulating magnetic topological transitions using antiferromagnetic order.

## Abstract (web summary)

Spin-polarized carriers could show an extra Hall component when moving through certain real-space topological spin textures. Here, He et al. report an exchange bias experienced by the topological spin textures living at the interface between a topological insulator and an adjacent antiferromagnet, suggesting a chiral spin texture is induced.

## Introduction

Spin-polarized carriers adiabatically moving through certain real-space topological spin textures can obtain a Berry’s phase as though they were in an applied magnetic field, resulting in a transverse carrier transport. Induced by this transverse transport, an extra Hall voltage can be observed, which is proportional to neither the applied external field nor the total magnetization. This spin-texture-induced extra Hall component is usually referred to as the topological or geometric Hall effect (GHE)^1^, which is a real-space counterpart of the *k*-space Berry phase in an intrinsic anomalous Hall effect (AHE). GHE is typically observed near magnetic reversal, within a window of the applied magnetic field and the temperature. Since the discovery of magnetic skyrmions in B20 compounds^2–5^ and heavy-metal multilayers^6–8^, GHE has been considered as an experimental signature of topological spin textures (such as skyrmions), and along with other real-space detection methods, it enables mapping of the magnetic topological phase diagram^9–12^. The topological charge of a real-space spin texture $\left[ {{\mathbf{S}}({\mathbf{r}})} \right]$ given by $N = \frac{1}{{4\pi }}\mathop {\int }\nolimits \mathrm{d}{\mathbf{r}}^2{\mathbf{S}} \cdot \left( {\partial _x{\mathbf{S}} \times \partial _y{\mathbf{S}}} \right)$ provides an emergent gauge field of one flux quantum (${{\noexpand\iPhi}} _0 = h/e$) experienced by the carriers passing through. Therefore, the magnitude of the GHE is proportional to the topological charge density. Recently, signatures of such GHE have been reported in thin film heterostructures composed of magnetically doped topological insulators (TIs) and intrinsic TIs^9^. Based on the theoretical calculations using a three-dimensional tight-binding model of this system, Néel-type skyrmions are shown to be energetically preferred over collinear spin textures, providing strong evidence of a skyrmion phase^9,13^.

In addition to the ferromagnet/TI interface, recent developments in topological antiferromagnetic (AFM) spintronics have demonstrated rich physics at the interface between a TI thin film and an AFM layer. At this interface, the surface Dirac fermions interact with the terminating magnetic atoms on the AFM surface, giving rise to interesting emergent phenomena, such as an interfacial ferromagnetic (FM) order^14–17^. Such FM order breaks the time-reversal symmetry of the TI surface, inducing a finite Berry curvature and resulting in an AHE. With this FM layer stabilized by the AFM layer, the proximity-induced AHE survives at a much higher temperature than that in typical magnetically doped TIs. This scenario requires the interfacial FM layer to strongly couple with the AFM layer, and has been experimentally observed^14^ through exchange bias of the AHE and a staggered magnetic switching in a magnetic TI/AFM heterostructure [Cr:(Bi,Sb)_2_Te_3_/CrSb].

Here, we report an experimental observation of the GHE modulated by uncompensated pinned spins in the AFM layer at the interface between an intrinsic TI thin film of (Bi,Sb)_2_Te_3_ and an AFM layer of MnTe. This suggests that a topologically nontrivial chiral spin texture is induced in the TI through interactions with the spin-polarized Mn planes of the MnTe. We find that the magnetic topological charge can be manipulated by a seeding effect of pinned spins in the AFM layer. Systematic experimental results of the carrier magnetotransport, neutron scattering, and magnetic X-ray absorption (XA) spectroscopy support that the interfacial FM layer is induced in the TI through proximity interactions with the AFM layer.

## Results

### Topological insulator and antiferromagnet heterostructures

**Fig. 1:**
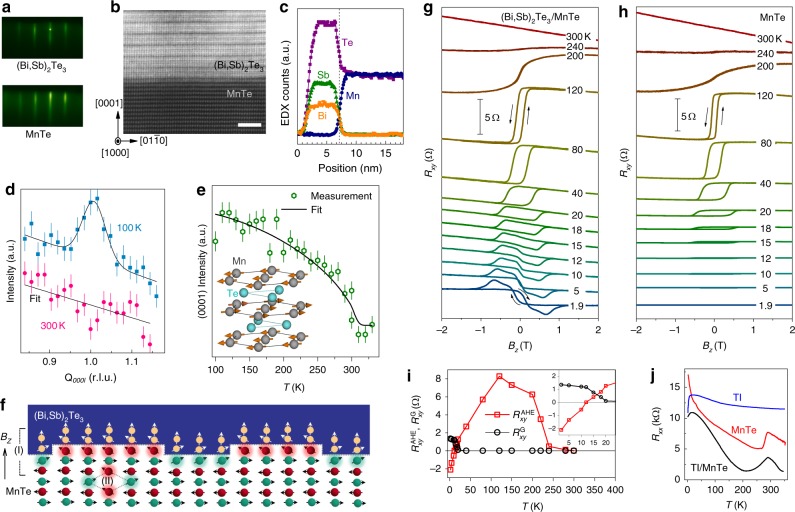
Emergent geometric Hall effect in a TI and antiferromagnet bilayer. **a** Reflection high-energy electron diffraction patterns of TI (Bi,Sb)_2_Te_3_ and antiferromagnet MnTe. **b** A high-resolution transmission electron microscopy image of the bilayer cross-section. The scale bar represents 2 nm. **c** An atomic-resolution energy-dispersive X-ray spectroscopy (EDX) line-scan across the bilayer interface, demonstrating the sharpness of the elemental profiles at this interface. **d** The neutron diffraction measurement of the MnTe (0001) peak at 100 and 300 K. **e** the fitted temperature-dependent peak height suggest a *T*_N_ of ~300 K, consistent with the one shown in (**d**). **f** Schematics illustrating the spins in the TI bottom surface, the interfacial FM layer, and the AFM spins in the bilayer under a perpendicular magnetic field. The polarized spins in the TI bottom surface is induced by the TI-AFM proximity (Region I), while some small magnetizations in the AFM layer may come from the defects in the bulk (Region II). **g** Field-dependent Hall resistance loops of the bilayer at different temperatures, which show a clear evolution from the AHE at high temperature to the GHE at low temperature. However, in a single MnTe layer, this GHE does not exist at the corresponding temperature range as shown in (**h**), demonstrating the role played by the top TI layer. **i** The magnitude of the AHE $(R_{xy}^{{\mathrm{AHE}}})$ and the GHE $(R_{xy}^{\mathrm{G}})$ at different temperatures extracted from (**g**). **j** The temperature-dependent longitudinal resistance of a pure TI, a MnTe layer, and the combined heterostructure. Error bars represent one standard deviation

The presence of an induced FM order at the TI/AFM interface is manifested as an AHE effect observed in the magnetotransport measurements. In this experiment, the TI thin film, (Bi,Sb)_2_Te_3_, was epitaxially grown on the top of a lattice-matched AFM, NiAs-phase MnTe^18^, using molecular beam epitaxy (see Methods). The crystal quality is evidenced by the streaky reflection high-energy electron diffraction (RHEED) patterns, whereas the quality of the interface can be seen in the high-resolution transmission electron microscopy image, as shown in Fig. 1a, b, respectively. An atomic-resolution energy-dispersive X-ray spectroscopy (EDX) line-scan was measured across the (Bi,Sb)_2_Te_3_/MnTe interface (Fig. 1c), demonstrating the sharpness of the elemental profiles. Bulk MnTe is known to be a semiconducting A-type antiferromagnet (Néel temperature *T*_N_ ~ 307 K), with parallel Mn spins in each basal plane. The spins between adjacent planes are antiparallel, as shown in Fig. 1d inset^18^. To confirm this magnetic order in the MnTe thin film, neutron diffraction experiments were carried out and the results are summarized in Fig. 1d, e. A (0001) peak (Fig. 1d) was observed as the signature of this magnetic order, which vanishes above *T*_N_ ~ 300 K (Fig. 1e), similar to the *T*_N_ of the bulk. A possible interfacial spin structure is shown in Fig. 1f, and is discussed in more detail below. The AHE is plotted in Fig. 1g, where a hysteretic AHE is captured by scanning the applied perpendicular magnetic field. Likewise, a control sample of a pure MnTe thin film is shown in Fig. 1h. Note that the linear component corresponding to the ordinary Hall effect is not subtracted from the measured data. When the applied field is large (e.g., above ±1 T), the $R_{xy}$ curve saturates to a line with a finite intercept at *B* = 0, indicating the sign and the magnitude of the AHE component. Below ~12 K, an unexpected transition occurs: the AHE component of $R_{xy}$ is negative for the positive field, and positive for the negative field. This polarity is opposite to those above 12 K, where the AHE has the same sign as the magnetization. To clearly demonstrate this trend, the magnitude of AHE ($R_{xy}$) is extracted from Fig. 1g as shown by the red curve in Fig. 1i. In addition to the change of sign, the magnitude of the Hall resistance surprisingly overshoots the saturated value, which is attributed to a GHE, as discussed below.

### Origins of anomalous Hall effects

The presence of two AHE components with opposite signs at different temperature ranges (Fig. 1g) suggests a significant change in charge transport scenario. To demonstrate this, the longitudinal resistance as a function of temperature $[R_{xx}\left( T \right)]$ was measured using control samples: a pure MnTe thin film, a pure TI thin film, and the combined heterostructure, as plotted in Fig. 1j. In this figure, the pure TI thin film (blue) exhibits metallic behavior below ~20 K, since the thermal broadening is narrow enough to exclude the scattering given by the bulk states so that the transport is dominated by the TI surface. On the other hand, MnTe (red) behaves as a semiconductor up to ~*T*_N_, and the resistance drops faster with increasing temperature compared to the TI layer. Therefore, it is likely that the TI surface dominates the low-temperature (<20 K) transport, whereas the MnTe layer dominates in high temperature.

The AHE contributions of the TI and MnTe layers are different, likely originating in interfacial proximity interactions and MnTe bulk defect spins, respectively. As illustrated schematically in Fig. 1f, since the spins of the front-most Mn atoms cannot be fully compensated, they are easy to tilt out-of-plane in an applied perpendicular field [highlighted near the interface in Region (I) of Fig. 1f]. These tilted surface spins may induce magnetic moments with *z* components on the TI side through exchange coupling (white arrows with beige spheres). In this way, a proximity-induced FM order on a TI surface will break the time-reversal symmetry and induce an intrinsic AHE^19–22^. The MnTe contribution likely arises from defects in the bulk MnTe that yield uncompensated spins [Region (II) in Fig. 1f], which can function as spin-dependent scattering centers, resulting in another AHE component. This is confirmed by referring to the data of the control sample prepared in the same condition, in which the AHE in a single MnTe layer does not change polarity (Fig. 1h). Note that the illustration in Fig. 1f suggests that both spin-sublattices can induce magnetization in the TI layer, and therefore the proximity-induced transport signatures can survive even with surface roughness and terraces. When the temperature is below 12 K, no AHE was observed from the control sample, suggesting that the negative AHE at *T* < 12 K observed in the TI/AFM heterostructure (Fig. 1g) was indeed associated with the TI layer.

As mentioned before, in the TI/AFM heterostructure, when *T* < 20 K, an extra GHE component appears right before the saturation of $R_{xy}$ during each magnetic reversal, suggesting the presence of topological spin textures such as skyrmions. For example, at 1.9 K (Fig. 1g), the field scan was initiated from +2 T along +*z*, where $R_{xy}$ is negative. As the field is swept to −*z* direction, $R_{xy}$ reverses at about −0.2 T and becomes positive. As the field along −*z* is increased further, $R_{xy}$ continues to climb and peaks at about −0.7 T, after which it drops to the value coinciding with that of a saturated magnetization at a large field, e.g., −2 T. Such a non-monotonic field dependence suggests the presence of a GHE. In Fig. 1i, the magnitude of this GHE is extracted from Fig. 1g at different temperatures, shown by the black curve. Since both the low-temperature AHE and the GHE signal occur only in the heterostructure, the GHE is likely induced at the TI/AFM interface as discussed before. Thus, the total Hall resistance can be considered to have three components: $R_{xy} = R_{xy}^0 + R_{xy}^{{\mathrm{AHE}}} + R_{xy}^{\mathrm{G}}$, where $R_0$ denotes the ordinary linear Hall resistance, $R_{xy}^{{\mathrm{AHE}}}$ is the anomalous Hall resistance, and $R_{xy}^{\mathrm{G}}$ is the geometric Hall component induced by topological spin textures. Here, $R_{xy}^{\mathrm{G}}$ is proportional to the ensemble average of the topological charge density 〈*N*〉, which is determined by the topological order of the real-space spin texture.

### Exchange-biased geometric Hall effects

**Fig. 2:**
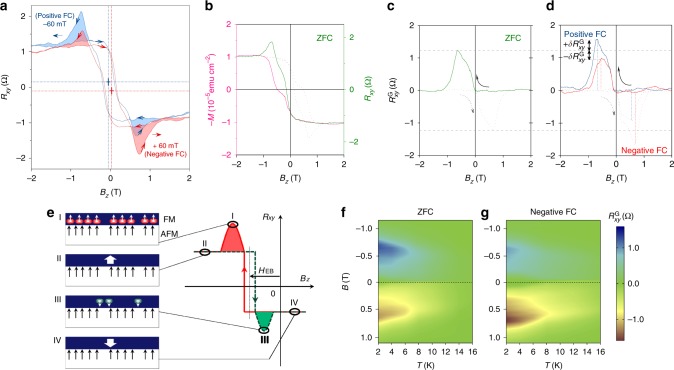
Exchange-biased geometric Hall effect (GHE). Shown are the data from the TI/antiferromagnet bilayer at 1.9 K. **a** Hall resistance $R_{xy}$ obtained in an applied perpendicular field. The dashed lines indicate the lateral shifts of $R_{xy}$. The colored areas show the magnitude change of the peak and dip regions referring to the GHE. This magnitude change along with the lateral shift is the signature of the exchange-biased GHE. **b** Plots of field-dependent magnetization and $R_{xy}$. The $M$–$B_z$ loop (pink) is obtained using a superconducting quantum interference device. The geometric Hall component, $R_{xy}^{\mathrm{G}}$, is obtained by subtracting the normalized magnetization (pink) from the $R_{xy}$ (green). **c** $R_{xy}^{\mathrm{G}}$ obtained after ZFC, whose magnitudes are compared with those obtained after positive/negative FCs processes as shown in (**d**), where $\delta R_{xy}^{\mathrm{G}}$ marks the differences. This magnitude change demonstrates the signature of the exchange bias experienced by the topological charges. The gray dashed lines are guides to the eyes. **e** A schematic demonstration of the exchange-biased topological charges. The anchoring spins in the AFM layer assist the nucleation of positive topological charges (red circles) while prohibit the negative ones (green circles). **f** Phase diagram of the GHE as functions of both the temperature and the magnetic field after ZFC. The blue and red areas show the $R_{xy(0)}^{\mathrm{G}}$ without the exchange bias, which is symmetric with respect to the $B = 0$ line. **g** The red $R_{xy}^{\mathrm{G}}$ component grows while the blue one shrinks after a negative FC process. Such an asymmetric phase diagram contrasts with the symmetric one obtained after a ZFC process, demonstrating the exchange bias experienced by the topological charges

More importantly, the observed magnitude of $R_{xy}^{\mathrm{G}}$ is modulated depending on the field-cooling (FC) direction, i.e., $R_{xy\left( \pm \right)}^{\mathrm{G}} = R_{xy\left( 0 \right)}^{\mathrm{G}} \pm \delta R_{xy}^{\mathrm{G}}$, where $R_{xy\left( 0 \right)}^{\mathrm{G}}$ is the GHE component obtained after zero FC (ZFC), + is for the positive FC, whereas − is for negative FC. This suggests that 〈*N*〉 can be exchange-biased by the pinning spins frozen after the FC: 〈*N*〉_±_ = 〈*N*〉_0_ ± *δ*〈*N*〉. As exhibited in Fig. 2a, this effect was captured by heating the heterostructure to 350 K and then cooled to 1.9 K in an applied field of 5 T along +*z* (positive FC) or −*z* (negative FC). Although the Néel vector of MnTe lies within the *x*–*y* easy plane, some AFM surface spins may still be canted and frozen after FC, contributing a perpendicular component, such that the interfacial FM layer experiences an exchange bias^23^ (Fig. 1f). Without the FC process, the *M*–*H* loops become symmetric with respect to the origin, and no indication of exchange bias was observed (Fig. 2b). Two steps occur during each magnetic reversal as shown by the pink curve in Fig. 2b, suggesting an intermediate metastable multi-domain phase. Here, the magnitude of $R_{xy}^{\mathrm{G}}$ can be extracted by subtracting the normalized field-dependent magnetization from the total $R_{xy}$^9,24^, as shown in Fig. 2c. When FC is applied, however, $R_{xy\left( \pm \right)}^{\mathrm{G}} = R_{xy\left( 0 \right)}^{\mathrm{G}} \pm \delta R_{xy}^{\mathrm{G}}$ occurs, as shown in Fig. 2d. Specifically, the positive FC (blue) enhances the magnitude of the GHE by $+ \delta R_{xy}^{\mathrm{G}}$ at −0.8 T, while it is reduced by $- \delta R_{xy}^{\mathrm{G}}$ (at 0.6 T). This scenario reverses in the case of negative FC (red). Measurements of GHE at different temperatures reveal the phase diagram of $R_{xy\left( - \right)}^{\mathrm{G}}$ as a function of the temperature and the applied field, as shown in Fig. 2f, g. Unlike the ZFC case in Fig. 2f, the FC phase diagram in Fig. 2g demonstrates the modulation of $- \delta R_{xy}^{\mathrm{G}}$. This $\delta R_{xy}^{\mathrm{G}}$component, and hence the topological-charge density 〈*N*〉, survives up to ~ 20 K (see Fig. 1i, inset). Although a simple picture of conventional exchange bias can explain the lateral shift of $R_{xy}^{\mathrm{G}}$ along the $x$ direction, it cannot explain the amplitude modulation after FC processes.

### Theoretical model of the seeding effect

**Fig. 3:**
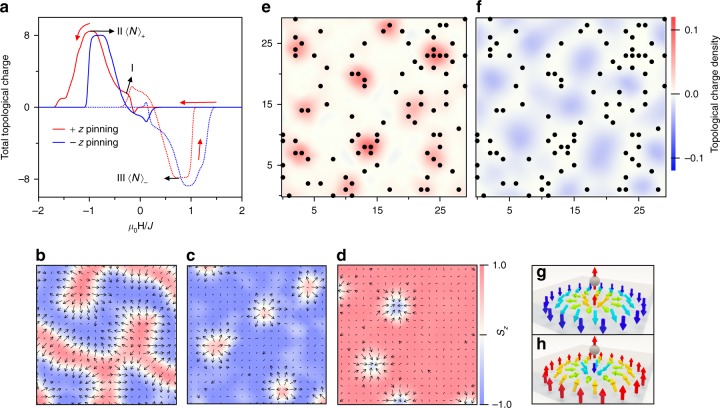
Stochastic micromagnetic simulation of the topological exchange bias. This simulation was calculated using a toy model on a 30 × 30 grid with a periodic boundary condition. The topological charges in this model are assumed to be carried by magnetic skyrmions. **a** The calculated total topological charge averaged during 300 cycles of the sweep of the applied field. The solid lines denote the positive-to-negative field scan, whereas the dotted lines denote the inverse ones. **b**–**d** Three snapshots of the simulation at different states of the switching: **b** is for a helical phase of stripe domains; **c** is for positive topological charges, and **d** is for negative ones. Averaged topological charge density for **e** positive and **f** negative topological charges. The anchoring spins are denoted by the circular solid points, with their spins polarized out of plane. The color map of the average local topological charge denotes the possibility of the topological-charge nucleation, red for positive charges and blue for negative ones. Opposite anchoring spins give rise to a similar picture but with the opposite sign. **g**, **h** Two different spin configurations with the topological charges changing polarities with respect to the frozen anchoring spins in the AFM layer. **g** Positive topological charges are formed with their central spins parallel with the anchoring spins, whereas (**h**) shows the case of an opposite alignment

This modulation of $\pm \delta R_{xy}^{\mathrm{G}}$ may stem from a seeding effect induced by the anchoring spins in the AFM layer frozen after the FC process. Experimentally, it has been shown that special domain nucleation patterns can be induced by the spins in an adjacent AFM layer^25^ due to interfacial exchange coupling. Here we show that this exchange coupling can result in a seeding effect for the spin-texture topology. The microscopic picture is schematically shown by the four scenarios illustrated in Fig. 2e. After positive FC, some pinned spins can be frozen in the AFM layer along the FC direction (black arrows) due to thermoremanence^26,27^. When the applied field sweeps to I, positive topological charges [red circles with up central spins in I] are created through interactions with the pinned spins. After the saturation along a negative field II, these positive topological charges are annihilated. When the field scans to III, negative topological charges are prohibited by the pinning spins and therefore are more likely to nucleate outside the spin-pinned regions, but with a lower density [green circles with down central spins in III]. These negative charges again vanish after magnetic saturation IV. The modification of the topological charge density during the above four scenarios can be described as 〈*N*〉_±_ = 〈*N*〉_0_ ± *δ*〈*N*〉. This results in a shift of the Hall signal: $R_{xy\left( \pm \right)}^{\mathrm{G}} = R_{xy\left( 0 \right)}^{\mathrm{G}} + \delta R_{xy}^{\mathrm{G}}$. A similar modulation of $- \delta R_{xy}^{\mathrm{G}}$ and −*δ*〈*N*〉 occurs after a negative FC. To further understand this effect, a micromagnetic simulation based on a toy model was carried out, mimicking the thin FM layer at the TI/MnTe interface. Anchoring spins were randomly generated in the simulation plane, with their spins$\left\{ {{\mathbf{S}}_{j_{{\mathrm{pin}}}}} \right\}$ fixed along either +*z* to mimic a positive FC or −*z* for a negative one. These anchoring spins interact with the spin texture $\left\{ {{\mathbf{S}}_i} \right\}$ through the exchange Hamiltonian $h_{{\mathrm{pin}}} = - J_0\mathop {\sum }\limits_{i,j_{{\mathrm{pin}}}} {\mathbf{S}}_i \cdot {\mathbf{S}}_{j_{{\mathrm{pin}}}}$. Dynamical behavior of the spin texture was obtained by solving Landau–Lifshitz–Gilbert equation, with a stochastic field describing the effect of the thermal fluctuation (see Methods). Although the magnetic topological charge can arise from different spin textures, in this model we use magnetic skyrmions for simplicity. The average total topological charge, 〈*N*〉, was tracked by averaging over 300 reversals of the external field, as shown in Fig. 3a. Snapshots during one of the 300 simulations are shown in Fig. 3b–d. When the anchoring spins are fixed along +*z*, the spin texture first presents a phase of helical domains (Fig. 3b), and then the helical domains break and shrink into individual positive skyrmions (Fig. 3c). When this occurs, 〈*N*〉 is maximized. As the field is increased along −*z*, Zeeman coupling dominates, annihilating all topological charges and resulting in a saturated FM order along −*z*. As the field is scanned back from negative to positive, the spin texture evolves in an analogous way, with the sign of 〈*N*〉 reversed (Fig. 3d). A movie recording this dynamical process among one of the 300 simulations can be found in Supplementary Movie 1. Strikingly, the modulation of the topological charge density is numerically captured, namely, 〈*N*〉_+_ = 〈*N*〉_0_ + *δ*〈*N*〉 for positive FC, whereas 〈*N*〉_−_ = 〈*N*〉_0_ − *δ*〈*N*〉 for negative FC, suggesting that the topological charge density is determined by the direction of the pinning spins with respect to the sign of the created topological charge. This is in agreement with our experimental results. To further demonstrate this point, using the +*z* pinning as an example, the averaged topological charge areal density, 〈$\rho \left( {\mathbf{r}} \right) = \frac{1}{{4\pi }}{\mathbf{S}} \cdot ( {\partial _x{\mathbf{S}} \times \partial _y{\mathbf{S}}} )$〉, is plotted in Fig. 3e, f, for the cases of II and III, respectively. The positions of the anchoring spins are shown by the solid black dots, whereas the color illustrates the map of 〈$\rho \left( {\mathbf{r}} \right)$〉. As shown by the topological charge density, +*z* anchoring spins assist in the nucleation of positive charges but prohibit negative ones. Induced by the exchange coupling $h_{{\mathrm{ach}}} = - J_0\mathop {\sum }\limits_{i,j_{{\mathrm{ach}}}} {\mathbf{S}}_i \cdot {\mathbf{S}}_{j_{{\mathrm{ach}}}}$, positive 〈*N*〉 are energetically preferred to coincide with +*z* anchoring spins, since the spin at each skyrmion core is parallel with the anchoring spin (Fig. 3g). Negative skyrmions, on the other hand, have their central spins anti-parallel with the anchoring spins, and therefore are more difficult to stabilize (Fig. 3h). Note that the creation and annihilation of a topological charge only involve several local spins^28^, therefore the seeding effect of topological charge creation in this simulation should also be expected in other more complex topological spin textures.

### Mechanisms of the exchange-biased topological charges

**Fig. 4:**
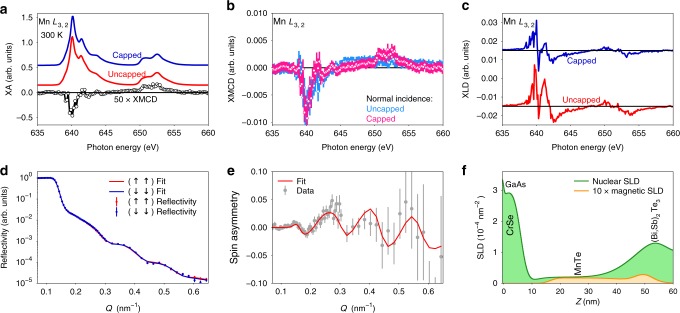
Probing the interfacial magnetism in the TI and antiferromagnet heterostructure. **a** Background-subtracted Mn *L*-edge XA spectra of a TI-capped (blue) and uncapped (red) MnTe films (300 K) alongside the representative normal incidence Mn *L*-edge XMCD of a capped sample (black) (15 K). The fact that the TI capping does not change the valence of Mn atoms from 2+ to 3+ suggests negligible Mn inter-diffusion. The spectra have been offset for clarity. **b** Comparison of Mn *L*-edge XMCD for capped and uncapped samples in normal incidence (15 K) probing the in-plane and out-of-plane ferromagnetic alignment of moments. The error bars are one standard deviation. **c** Antiferromagnetic order probed by XLD. Data give a comparison of Mn *L*-edge XLD for both capped and uncapped samples. The XMCD and XLD data suggest that the AFM order of the MnTe is not altered by the TI capping layer. **d** Fitted polarized neutron reflectometry of the TI/MnTe heterostructure measured at 7.5 K in a 700 mT in-plane applied field. **e** Spin asymmetry of the measurement with the fit shown in (**d**). Error bars represent one standard deviation. **f** Structural and magnetic depth profile are represented by the nuclear and magnetic scattering length densities (SLD) used to obtain the fit shown in (**d**) and (**e**). These polarized neutron reflectometry results (**d**, **e**, and **f**) suggest an interfacial ferromagnetic layer was induced in the TI/AFM heterostructure

An important question in the observed exchange-biased GHE is the origin of the induced FM layer at the TI/AFM interface. This layer could be induced either by the proposed magnetic proximity effect, or by the migration of enough Mn from the AFM layer into the TI layer. The latter would result in a magnetically doped TI, Mn_*δ*_(Bi,Sb)_2−*δ*_Te_3_, where the observed 20 K onset temperature of the GHE would require that *δ* is significantly in excess of 0.15 in order to achieve the necessary Curie temperature^29,30^. Such doping-induced ferromagnetism would be accompanied by an increase in the Mn valence from 2+ toward 3+ and a significant enhancement of the X-ray magnetic circular dichroism (XMCD) magnitude^31,32^. We therefore probed Mn valence and magnetic ordering using total electron yield (TEY) soft XA spectroscopy and XMCD, shown in Fig. 4a, b. When using the TEY mode, the probing depth is approximately 5 nm^33^, increasing sensitivity to the near-surface Mn in a TI(3 nm)/MnTe heterostructure. Figure 4a shows the characteristic Mn *L*-edge XA spectra of two MnTe films: One with and another without the TI capping layer (blue and red curves, respectively). The spectra are consistent with reported results of MnTe and exhibit Mn^2+^ lineshapes both before and after the TI capping^34–36^, indicating that the TI layer does not alter the valence of interfacial Mn atoms in MnTe and that Mn diffusion into the TI is negligible. The presence of a vanishingly small magnetic polarization was captured by normal incidence XMCD measurements at the Mn *L*_3,2_ edge, as shown in Fig. 4b. The XMCD lineshape is again consistent with past examples of FM Mn^2+^ in MnTe and Ge_1−*x*_Mn_*x*_Te^34–36^, where the *L*_3_ edge of the MnTe and MnTe/TI films exhibit negative peaks at 640.3 ± 0.1 eV and 640.1 ± 0.1 eV, respectively. Thus, no signs of energy shifts associated with valence changes are observed.

In addition, the XMCD evaluation of Mn magnetizations for the TI-capped and uncapped samples further excludes inadvertent Mn-doping into the TI layer. On one hand, the detected *L*_3_ XMCD ratio is *R*_XMCD_ = (XMCD_capped_/XMCD_MnTe_) = 0.972 ± 0.056. Fitting of the dichroism peak areas and analysis of the integrated dichroism under the *L*_3_-edge suggest that the capped sample has the same Mn-moment as that of the uncapped, which places an upper bound on the total magnetization, *M*_Capped_ < 1.2 × *M*_Uncapped_ (99.997% confidence). On the other hand, recent studies have shown that Mn-doping in FM TI films would result in XMCD values of 75–125% at the Mn *L*_3_ edge^31,32^, while our capped film exhibits an XMCD of only 0.73 ± 0.04%, two orders of magnitude lower. Comparison with reference spectra in the literature yield an estimated magnetization of 0.02–0.04 *µ*_B_/Mn (3.5–7.5 e.m.u. × cm^−3^; 1 e.m.u. × cm^−3^ = 1 kA × m^−1^)^35^. Such small magnetizations are not likely induced by Mn doping, and is consistent with the weak AHEs in Fig. 1g, h.

Moreover, the AFM orders in both the capped and uncapped MnTe samples were probed by the X-ray linear dichroism (XLD) measurements, as shown in Fig. 4c. Here also, the peak locations are consistent with each other, suggesting that the charge anisotropy and the AFM ordering are likely undisturbed after capping with the TI layer. The TI- and MnTe-thickness dependences experiments by independently controlling the thickness of each layer (Supplementary Figs. 1, 2) also support this conclusion (Supplementary Note 1).

Having established the origin of the observed interfacial magnetism, its contribution to magnetization was directly probed using polarized neutron reflectometry (PNR). PNR was performed on a 42 nm-MnTe film capped with a 9 nm-TI layer after FC to 7.5 K under an in-plane applied field of 700 mT. The fitted reflectivity of this sample, the associated spin asymmetry, and the best-fit depth profile are shown in Fig. 4d–f, respectively. We detected a small net magnetization of approximately 10–20 e.m.u. × cm^−3^ (95% confidence interval) in the TI layer near the interface while a trace magnetization of approximately 6 e.m.u. × cm^−3^ can be seen within the MnTe layer in Fig. 4f, in good agreement with the estimations from XMCD. We note that the broadened TI/MnTe interface shown by the scattering length densities (SLD) nuclear profile is attributed to the terraces and long-range surface roughness typically show up in epitaxial TI growth, and does not indicate intermixing^37–39^. The magnitude of the induced moment at the interface is comparable to other examples of interfacial proximity interactions reported in the literature^40,41^. Note that alternative models (Supplementary Figs. 3, 4), which do not incorporate an interfacial magnetization in the TI, fail to describe the data. For more information on the PNR fitting process and alternative models, see Supplementary Note 2.

In summary, we have experimentally demonstrated the seeding of topological charges at a TI/AFM interface induced by the pinning spins. The exchange-biased topological charges are experimentally observed as a modulation of the GHE magnitude captured in a transport measurement under different FC conditions. This suggests that the creation of topological charges in a FM spin texture can be strongly affected by an adjacent antiferromagnet. Further X-ray and neutron experiments suggest that this FM order is likely induced by the proximity effect, rather than unintentional Mn migration.

## Methods

### Growth of magnetic heterostructures

All heterostructures in this work were fabricated on epi-ready semi-insulating GaAs(111)B substrates in an ultrahigh vacuum molecular beam epitaxy system. Prior to sample growth, oxide-desorption processes were carried out at 580 °C for 30 min. A thin insulating NiAs-type CrSe layer serves as a buffer for the growth of the MnTe layer. The substrate temperature was then maintained at 380 °C during the growth of MnTe and 200 °C for (Bi,Sb)_2_Te_3_. High-purity Mn, Bi, and Cr were evaporated from standard Knudsen cells, whereas Se, Sb, and Te were evaporated by standard thermal cracker cells. Real-time RHEED (as shown in Fig. 1a) was used to monitor all the growth cycles. The growth conditions of CrSe, MnTe, and (Bi,Sb)_2_Te_3_ layers were optimized such that very sharp, smooth, and streaky RHEED patterns were obtained.

### Neutron diffraction

High-angle neutron diffraction measurements were performed on the BT-4 triple axis spectrometer at the NIST Center for Neutron Research. Measurements were carried out in a temperature range of 100–325 K in a closed-cycle refrigerator. The incident and scattering neutron energy was 14.7 meV (*λ* = 2.359 Å), selected by pyrolitic graphite (PG) monochromator and analyzer crystals with multiple PG filters before and after the sample to eliminate higher-order neutrons. The Soller collimator configuration in downstream order was open-monochromator-20′-sample-20′-analyzer-open-detector. Measurements were taken by scanning the momentum transfer vector along the (000l) direction at the expected AFM peak location. In this configuration, the neutrons are exclusively sensitive to magnetic moments within the film plane, so that the magnetic order observed in this peak location suggests commensurate AFM ordering of in-plane spins.

### Polarized neutron reflectometry

PNR was performed on the PBR instrument at the NIST Center for Neutron Research. Samples were cooled to a temperature of 7.5 K in an in-plane applied field of 700 mT. Incident and scattered neutrons were spin-polarized either spin-up (↑) or spin-down (↓) with respect to the applied magnetic field. Two spin-flippers and polarizing supermirrors were used to select the incident and scattered neutron polarization direction. Thus, we measured the reflectivities as a function of the momentum transfer along the film normal direction in a range of 0.1–0.6 nm^−1^. The neutron propagation direction was perpendicular to both the sample surface and the applied field direction. Based on magnetometry measurements showing in-plane magnetization saturation below the applied field of 700 mT, spin-flip scattering is not expected. Therefore, we refer exclusively to the spin-up and spin-down non-spin-flip reflectivities, which are functions of the nuclear and magnetic scattering length density profiles. The nuclear and magnetic profiles were deduced through modeling of the data with the NIST Refl1D software package. The DREAM algorithm implemented in the Refl1D software is used to estimate the 95% confidence interval of the TI magnetization, in which Markov-chain Monte Carlo fitting is adopted to estimate uncertainties and yields a 95% confidence interval for the magnetic SLD.

### Magnetoelectric transport measurement

Hall bar devices with dimensions of 20 μm × 30 μm were fabricated using standard photolithography for the transport measurements. Systematically altering experimental variables such as temperature, magnetic field, and frequency, in addition to multiple lock-in amplifiers and sourcemeters enable comprehensive and high-sensitivity transport measurements in all the devices.

### Toy model of the topological exchange bias

The toy Hamiltonian describing the interfacial FM layer is

$$\begin{array}{l}H = - J_{\mathrm{H}}\mathop {\sum }\limits_{i,j} {\mathbf{S}}_i \cdot {\mathbf{S}}_j + D\mathop {\sum }\limits_{i,j} \left( {{\hat{\mathbf z}} \times {\hat{\mathbf r}}_{ij}} \right) \cdot \left( {{\mathbf{S}}_i \times {\mathbf{S}}_j} \right)\\ -\mu _0\mathop {\sum }\limits_i {\mathbf{H}} \cdot {\mathbf{S}}_i - J_0\mathop {\sum }\limits_{i,j_{{\mathrm{pin}}}} {\mathbf{S}}_{j_{{\mathrm{pin}}}} \cdot {\mathbf{S}}_i,\end{array}$$

where $J_{\mathrm{H}}$ is the Heisenberg exchange coupling between nearest neighbors, $D$ denotes the DMI magnitude, ${\mathbf{H}}$ is the applied field, and $J_0$ is the exchange coupling between the pinning spins $\{ {{\mathbf{S}}_{j_{{\mathrm{pin}}}}} \}$ and the spin texture, $\left\{ {{\mathbf{S}}_i} \right\}$. $\frac{D}{{J_{\mathrm{H}}}} = 1$ and $\frac{{J_0}}{{J_{\mathrm{H}}}} = 0.8$ are applied as an example. The spin dynamical process was obtained by solving Landau–Lifshitz–Gilbert equation ${\dot{\mathbf S}} = - \gamma {\mathbf{S}} \times {\mathbf{H}}_{{\mathrm{eff}}} + \alpha {\mathbf{S}} \times {\dot{\mathbf S}}$, where $\gamma$ is the gyromagnetic ratio, and $\alpha = 0.1$ is the damping coefficient. The effective field is ${\mathbf{H}}_{{\mathrm{eff}}} = - \frac{{\partial H}}{{\partial {\boldsymbol{S}}}} + {\mathbf{L}}$, where ${\mathbf{L}}$ is the stochastic field satisfying dissipation–fluctuation relation 〈$L_\mu \left( {{\mathbf{r}},t} \right)L_\nu \left( {{\mathbf{r}}\prime ,t\prime } \right)\rangle = \xi \delta _{\mu \nu }\delta _{rr\prime }\delta _{tt\prime }$ ($\mu ,\nu = x,y,z$), and $\xi = \frac{{\alpha k_{\mathrm{B}}T}}{\gamma }$. Here, $\frac{{k_{\mathrm{B}}T}}{{J_{\mathrm{H}}}} = 0.3$ is used as an example to mimic the thermal fluctuation given by the finite temperature.

### X-ray absorption spectroscopy and dichroisms

X-ray spectroscopy at the Mn *L*_3,2_ absorption edge (635–660 eV) was performed using TEY as the detection mode at beamline 4.0.2 of the Advanced Light Source at Lawrence Berkeley National Lab. All XA spectra were normalized to the intensity of the non-resonant pre-edge region; i.e., at energies below the *L*_3_ absorption edge. XMCD and XLD spectra were further normalized to allow direct comparisons, by shifting the pre-edge intensity to 0 and then scaling to set the polarization-averaged height of the most intense peak to 1.

Induced FM moments were probed by XMCD, in which photon energy scans were measured with 90% circularly polarized X-rays in applied magnetic fields of ±0.4 T. The XMCD was obtained as the difference between scans with opposite magnetic fields, and confirmed by also reversing the circular polarization. The X-ray beam was oriented along either a 30° grazing incidence or at (90°) normal incidence to probe in-plane or out-of-plane FM moments, respectively.

XLD was utilized to probe AFM order by measuring linear dichroism at 15 and 300 K. Without any structural phase transition in that range, the crystal field effects are presumed to be constant vs. temperature, so that the difference in dichroism can be attributed mainly to the magnetic contribution. XLD was measured in the 30° grazing incidence configuration using linearly polarized X-rays, and by rotating the linear polarization of X-rays between two orthogonal settings: fully in-plane (vertical polarization) and 30° from the film normal (horizontal polarization).

### Data availability

The data that support the plots within this paper and other findings of this study are available from the corresponding author upon reasonable request.

## Electronic supplementary material

Supplementary Information

Description of Additional Supplementary Files

Supplementary Movie 1
