# Supplementary material for: Exchange-biasing topological charges by antiferromagnetism
Source: Nat Commun. 2018 Jul 17;9:2767. doi: 10.1038/s41467-018-05166-9 (PMC6050290; doi:10.1038/s41467-018-05166-9)
Supplement: Supplementary file 1 — Supplementary Information [file 41467_2018_5166_MOESM1_ESM.pdf]

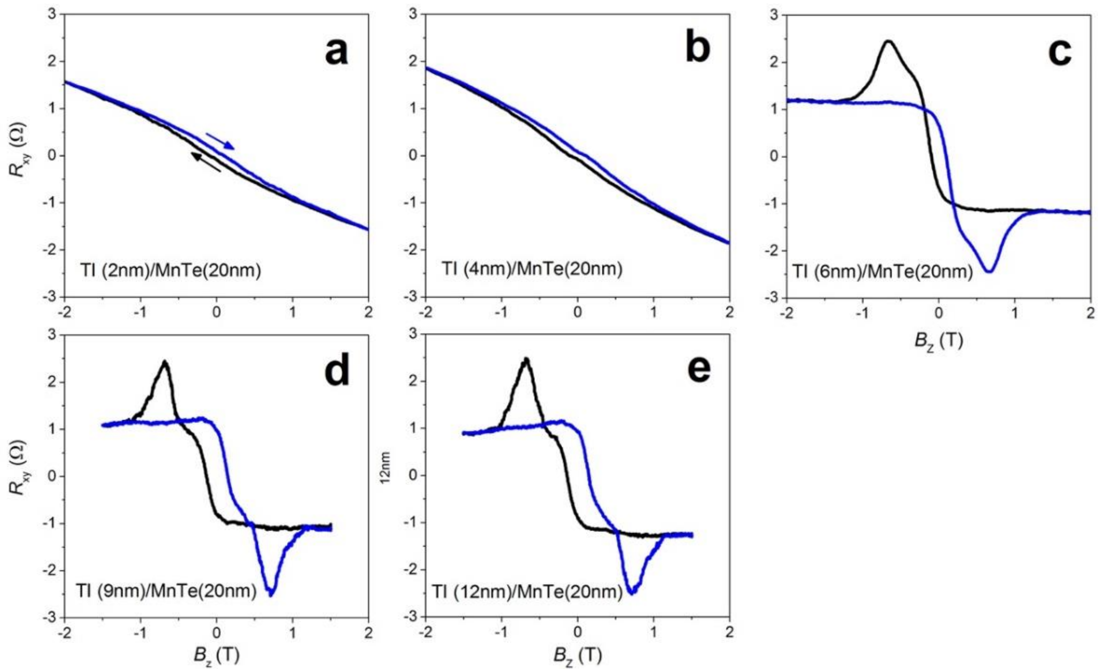

Supplementary Figure 1. Hall resistance measurements on TI/MnTe heterostructures with different TI thicknesses and fixed MnTe thickness. The MnTe thickness is fixed to 20 nm while the TI thicknesses are **a**. 2 nm, **b**. 4 nm, **c**. 6 nm, **d**. 9 nm, and **e**. 12 nm, respectively.

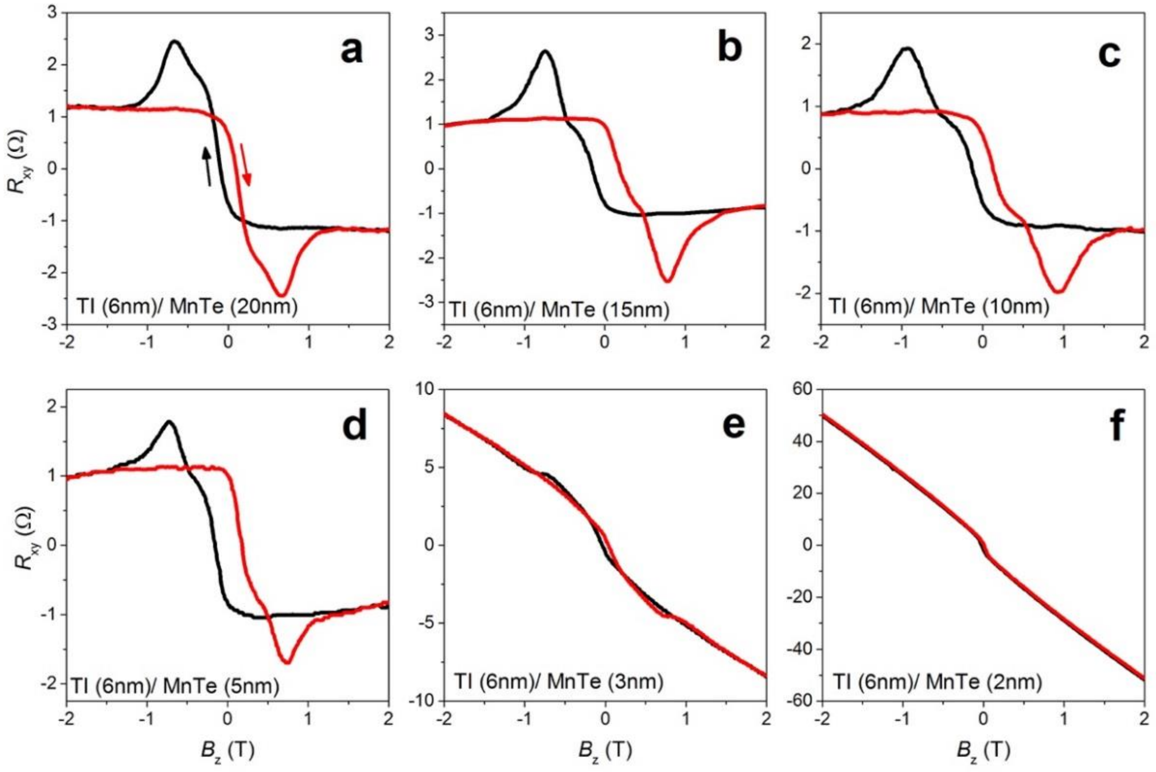

Supplementary Figure 2. Hall resistance measurements on TI/MnTe heterostructures with different MnTe thicknesses and fixed TI thickness. The TI thickness is fixed to 6 nm while the MnTe thicknesses are **a**. 20 nm, **b**. 15 nm, **c**. 10 nm, **d**. 5 nm, **e**. 3 nm, and **f**. 2 nm, respectively.

Supplementary Note 1: Thickness dependence of the magnetotransport

To understand the role of the two layers in heterostructure in the observed magnetotransport behavior, we have carried out thickness-dependent measurements, varying the thickness of either the TI layer or the MnTe layer, as shown in Supplementary Figures 1 and 2. Here, the Hall resistance is measured from two series of heterostructures:

one is varying the TI thickness from 2 nm to 12 nm with fixed MnTe thickness to 20 nm (Supplementary Figure 1), whereas the other one is varying the MnTe thickness from 2 nm to 20 nm with fixed TI thickness to 6 nm (Supplementary Figure 2). Based on these results, it seems both the MnTe layer and the TI layer should be thick enough to host the observed transport signatures. On one hand, if the TI layer is too thin, the hybridization between the top and the bottom surfaces open a gap, which might overwhelm the small exchange gap due to the magnetic proximity, which prohibits the Berry curvature and thereby rules out the AHE. On the other hand, if the MnTe layer is too thin, the magnetic order cannot be maintained, therefore the proximity effect is eliminated.

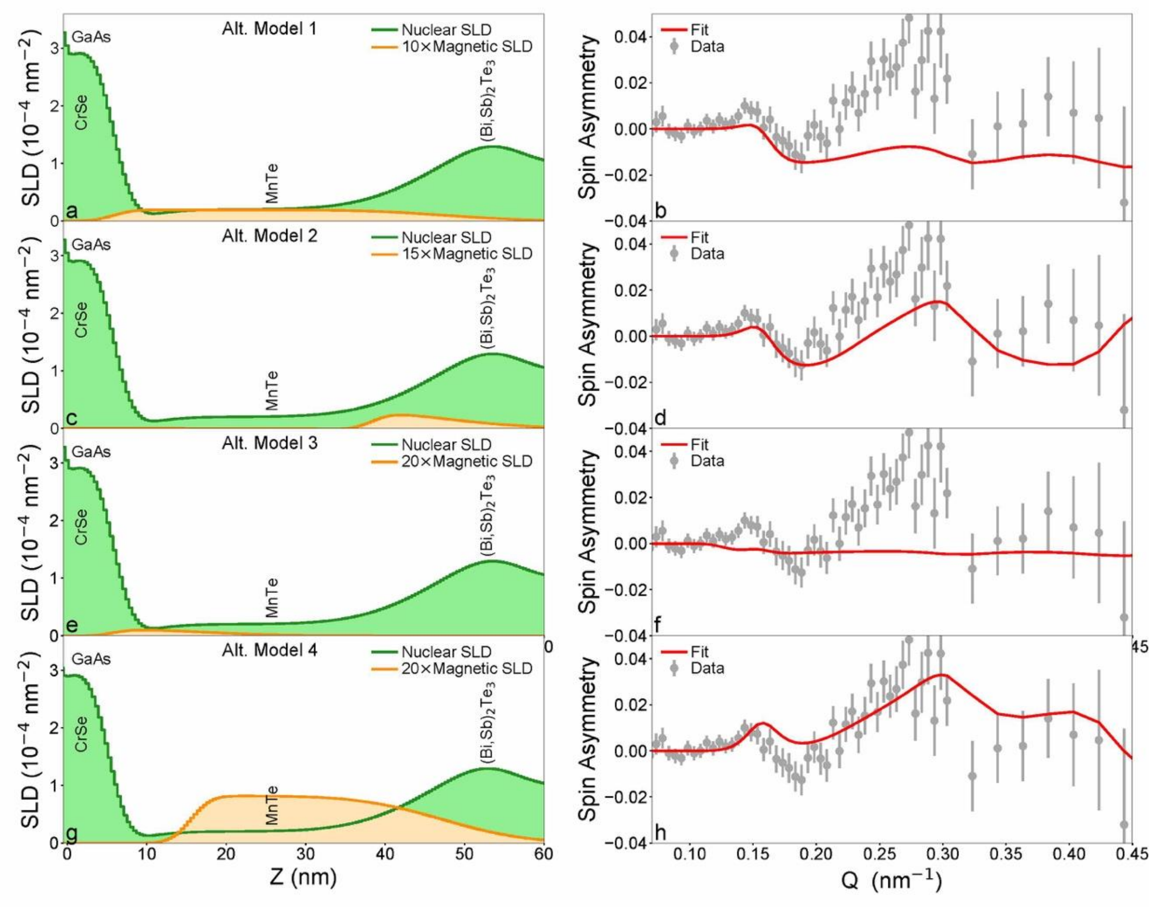

Supplementary Figure 3. Alternative Models 1-4 fitted to the PNR data of a TI/MnTe heterostructure (at 7.5 K with a 700 mT in-plane field). **a**, **c**, **e**, and **g** show the structural and magnetic scattering length densities (SLDs) of the four models, respectively. **b**, **d**, **f**, and **h** show the detailed spin asymmetry with these models fitting, respectively. Error bars represent one standard deviation.

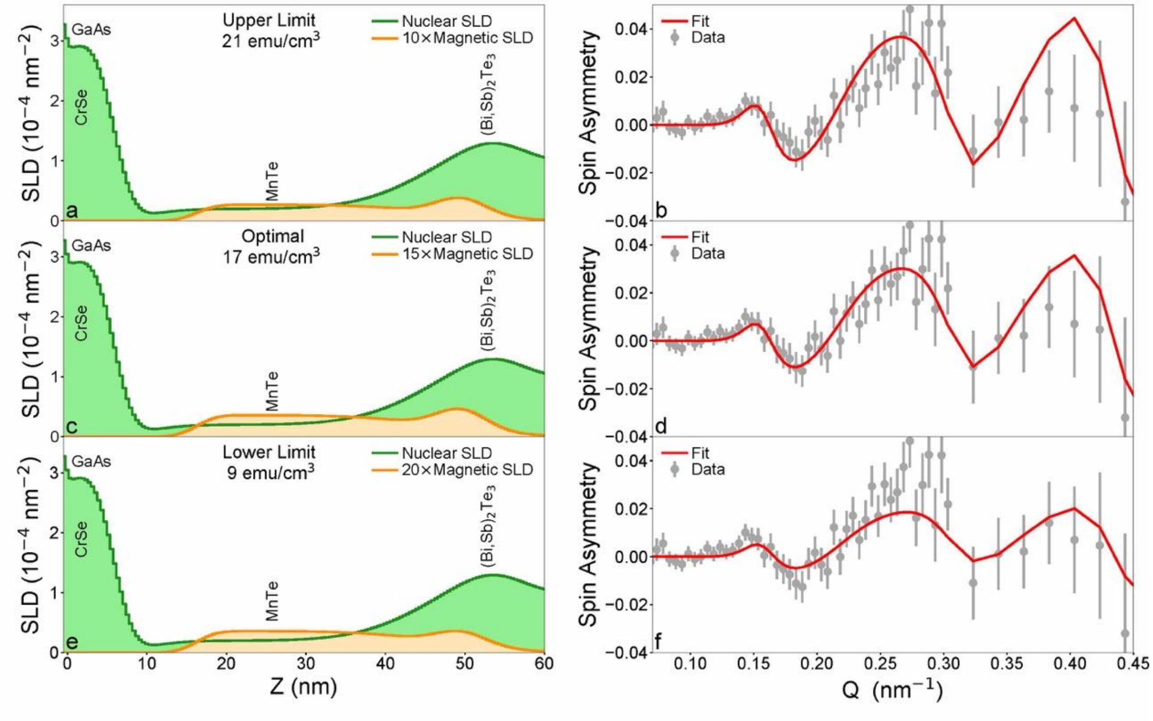

Supplementary Figure 4. Three models used to estimate the upper and lower bounds for TI magnetization. **a**, **c**, and **e** show the structural and magnetic scattering length densities (SLDs) of the three models, respectively. **b**, **d**, and **f** show the detailed spin asymmetry with these models fitting. Error bars represent one standard deviation, respectively.

Supplementary Note 2: Polarized Neutron Reflectometry Fitting and Alternative Models

Fitting of the PNR data was accomplished using the Refl1D software package for χ^2 minimization, while the uncertainty in the extracted magnetization was characterized using the DREAM algorithm implemented through the BUMPS python package. DREAM uses Markov-chain Monte Carlo fitting to estimate uncertainties, and yields a 95% confidence interval of (0.027-0.051)×10-4 nm-2 for the magnetic SLD in the TI at the interface, which

corresponds to a magnetization range of 10-20 emu/cc. However, examining such small moments with PNR often requires long counting times and good statistics near the critical edge. In this way, the magnetic signal is vanishingly small compared to the nuclear structure and may not significantly drive the fitting engine. Therefore we have examined a number of alternative depth profiles to identify any physical models which may be able to describe the data without a magnetized TI interface. Each model was allowed to optimize within a relatively wide range parameter space, in order to explore a wide range of structural and magnetic depth profiles. As expected, all of these models converged to essentially the same nuclear profile, so that only the resulting magnetic depth profile varies between fitted models. As shown in Supplementary Figure 3 below, we examined the following alternative models which do not allow for any interfacial magnetization within the TI layer:

First, MnTe is uniformly magnetic, TI is nonmagnetic; second, MnTe is magnetic with a magnetic dead layer at the CrSe/MnTe interface. This dead layer is allowed to encompass the majority of the MnTe layer to account for possible solutions in which the bulk MnTe is nonmagnetic while interfacial intermixing stabilizes FM near the TI/MnTe surface; third, MnTe is magnetic with a magnetic dead layer at the TI/MnTe interface; fourth, MnTe is magnetic with both top and bottom dead layers.

Unless otherwise noted, the error bars in all figures represent one standard deviation. These models fail in the following ways, respectively:

For the first model, the initial spin asymmetry peak clearly too small. It requires that the spin asymmetry be negative near 0.25 nm^{-1} while a clear positive peak is actually observed. For the second model, large lower magnetic dead layers are unable to

simultaneously describe the spin asymmetry peaks as 0.145 nm^{-1} and 0.18 nm^{-1}. One is too large and the other too small. Further, the peak at 0.25 nm^{-1} is missed completely. For the third model, large upper magnetic dead layers completely fail to describe the magnetic features over all regions of Q. For the fourth model, even optimizing both top and bottom magnetic dead layers fails to describe the first (positive) and second (negative) spin asymmetry peaks.

In addition to using the standard 95% confidence interval to characterize the uncertainty, we also used alternative models to estimate the upper and lower limit of the total magnetization by optimizing a model with some magnetization induced in the TI layer. As shown in Supplementary Figure 4, we find an upper limit of 21 emu/cc, where spin asymmetry is found to be slightly too high at 0.18 nm^{-1} and 0.25 nm^{-1}, and a lower limit of 9 emu/cc, which results in a spin asymmetry slightly too small on all 3 peaks below 0.3 nm^{-1}. An optimized model is obtained with a magnetization of 17 emu/cc.

As shown in these results, the modeling is indeed quite sensitive to the magnetization at the TI/MnTe interface.
