# Supplementary material for: Exchange-biasing topological charges by antiferromagnetism
Source: Nat Commun. 2018 Jul 17;9:2767. doi: 10.1038/s41467-018-05166-9 (PMC6050290; doi:10.1038/s41467-018-05166-9)
Supplement: Supplementary file 2 — Description of Additional Supplementary Files [file 41467_2018_5166_MOESM2_ESM.pdf]

Description of Additional Supplementary Files

File Name: Supplementary Movie 1
Description: Supplementary Movie 1 demonstrates a typical micromagnetic dynamical process during one scan, out of the total 300 scans, of the applied magnetic field. Positive and negative topological charges nucleate after helical domains are formed. Due to the stochastic field given by thermal fluctuation, the possibility to create topological charges has a real-space distribution. The average of topological density among the 300 scans are shown by the colour plot in Figs. 3e and f in the main text.
