# Supplementary figures and images for: Exchange-biasing topological charges by antiferromagnetism
Source: Nat Commun. 2018 Jul 17;9:2767. doi: 10.1038/s41467-018-05166-9 (PMC6050290; doi:10.1038/s41467-018-05166-9)

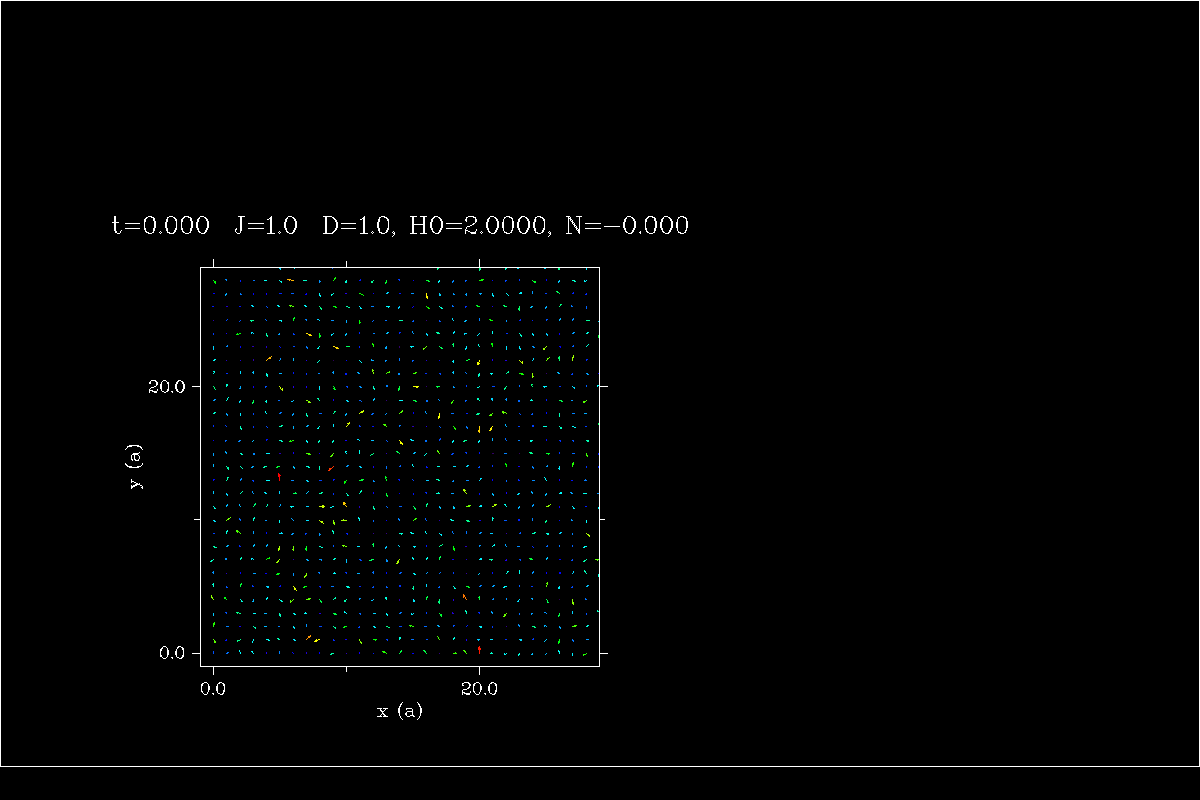

Supplement: Supplementary file 3 — Supplementary Movie 1 [file 41467_2018_5166_MOESM3_ESM.gif]
